# Menstrual hygiene among adolescent girls in junior high schools in rural northern Ghana

**DOI:** 10.11604/pamj.2020.37.190.19015

**Published:** 2020-10-29

**Authors:** Maxwell Tii Kumbeni, Easmon Otupiri, Florence Assibi Ziba

**Affiliations:** 1 Nabdam District Health Directorate, Ghana Health Service, Nangodi, Ghana,; 2 School of Public Health, Kwame Nkrumah University of Science and Technology, Kumasi, Ghana,; 3 Department of Nursing, University for Development Studies, Tamale, Ghana

**Keywords:** Adolescent girls, menstrual hygiene, management, sanitary products

## Abstract

**Introduction:**

the issue of menstrual hygiene is inadequately acknowledged and efforts to address the gaps has been unsatisfactory. Hygienic menstrual practice such as the use of sanitary pads is crucial during menstruation. Lack of sanitation facilities, especially for school girls, makes them vulnerable to emotional and physical challenges during their menstrual days. This study sought to investigate menstrual hygiene management among adolescent girls in junior high schools in rural northern Ghana.

**Methods:** a school-based cross-sectional study design was used. Multistage sampling technique was employed to select 730 school girls who had attained their menarche. Menstrual hygiene management was rated using the Selvi and Ramachandran scale. Bivariate analysis was conducted to compare good and poor menstrual hygiene management. The data were analyzed using STATA version 13.1.

**Results:**

the prevalence of good menstrual hygiene was 61.4%. Mothers' education and parents' socio-economic status were significantly associated with menstrual hygiene management. Inadequate sanitation facilities was a major challenge to menstrual hygiene management at schools. The use of sanitary pads was significantly associated with school attendance (p-value < 0.0001).

**Conclusion:**

the level of menstrual hygiene among in-school adolescent girls in northern Ghana is described as average. Although most of the schools had toilet facilities, they lacked clean water, soap, privacy and dustbins which are necessary for menstrual hygiene management. Interventions should target improving water, sanitation and hygiene facilities in schools as well as supply of pads to girls in rural school.

## Introduction

The World Health Organization (WHO) defines adolescence as persons from ten to nineteen years of age [1]. Adolescence is a crucial time period that comes with identity formation as well as transformation from childhood to adulthood [2]. This transformation is usually characterized by physical, psychological, mental, and social changes which are important to the adolescent´s wellbeing. These changes pose major challenges to parents and the growing adolescents [3]. Adolescence in females is usually marked with the onset of menses and this is seen as an important stage in their lives that requires special attention [4]. Unfortunately, issues concerning menstruation and menstrual hygiene are usually tagged with certain taboos and socio-cultural restrictions which have significantly altered the scientific understanding of menstruation and menstrual hygiene [4-6]. Good menstrual hygiene practice is a major issue facing adolescent girls especially those in the rural communities in sub-Saharan Africa. Studies from the African and the Asian continents have demonstrated unsatisfactory menstrual hygiene management among school-going girls [5,7]. It is estimated that about 200 million women and girls from developing countries struggle on daily basis during their menstrual period to get access to clean water for washing, and as well as convenient places for changing their pads [8]. Access to sanitary material has been a major challenge to menstruating women and girls as most of them use unhygienic absorbents during their menses [9-13]. Adolescent girls stay out of school during their menses because of lack of sanitary pads, and also lack of basic sanitation facilities to meet their privacy requirements [9,14].

A pilot study carried out in Ghana in selected senior high schools identified counselling of adolescent girls on puberty, and the provision of sanitary pads as a way to improving their school attendance. The study also identified factors such as poverty, non-availability of water and toilet facilities, which are typical of rural schools, to contribute negatively to school attendance among adolescent girls during their periods [15]. A case-control study in Ghana found that, discussing menstrual hygiene issues in class made girls appeared more confident and participated actively in such discussions. This improved their menstrual hygiene knowledge level and practices. The control group had difficulties managing their menses because teachers were not involved and issues about menstrual hygiene management were not also discussed in class [16]. Menstruation is a major cause of school absenteeism and school dropout rate among girls in rural areas. This negates the achievement of quality education (Sustainable Development Goal 4) among that age group. Taboos and socio-cultural restrictions on menstruating girls such as staying indoors, not going to pray at the mosque among others, are all against attaining gender equality (Sustainable Development Goal 5). These restrictions do not allow females to partake fully in their responsibilities when they are in their menstrual periods. Where there are no proper disposal facilities, there is likely to be indiscriminate dumping of used sanitary products that will then lead to environmental pollution hence achievement of the clean water and sanitation (Sustainable Development Goal 6) may be threatened [17]. The inability of adolescents to observe good menstrual hygiene practices is a major determinant of diseases and other complications among their age group [18]. Menstrual hygiene among girls in Ghana has not been given the needed attention by Government and Non-Governmental Organizations so far. This could be due to the fact that little research has been conducted in Ghana to provide information on menstrual hygiene among adolescent girls. This study investigated menstrual hygiene among adolescent girls in junior high schools in the Talensi district of Upper East Region, Ghana. The study assessed issues relevant to self-care practices, and barriers to menstrual hygiene among school-going adolescent girls.

## Methods

**Study design and setting:** a school-based analytical cross-sectional study was conducted in the Talensi district of Upper East region, Ghana. It was carried out from July to September 2016.

**Study population:** the study was conducted among junior high school adolescent girls aged 10-19 who had experienced menarche.

**Sample size and sampling strategy:** the sample size for the study was estimated using Epi Info Version 6. The total target population in all the junior high schools was 2329. The prevalence of poor menstrual hygiene practices was not known; therefore a 50% prevalence rate was used with 95% confidence interval and 5% margin of error. The estimated sample size was 330. This was multiplied by 2 to get 660 respondents in order cater for the design effect since a two-stage cluster sampling method was used. A 10% non-response rate of 66 was added to make 726 respondents. This was rounded up to a total sample of 730. The district has three sections: Central, West and East. Five (5) schools from each section were randomly selected, making a total of 15 schools for the study in the first stage of sampling. Then at stage two, 730 respondents aged 12-19 and who have attained menarche, were selected using simple random sampling with proportionate allocation of size based on the list of female students in the selected schools. Respondents were kept in spacious classrooms to ensure independent responses without any influence from colleagues.

**Data collection instrument and procedures:** structured questionnaire with closed and open-ended questions were administered to all study participants. The questionnaire was self-administered but was explained in the local dialect (Talen) by trained data collectors to the girls for a better understanding. A checklist was also used to assess the availability of menstrual hygiene-relevant sanitation facilities in the schools.

**Data quality management:** data quality was assured through careful design of the questionnaire. The tool was pretested in a nearby district that has characteristics like the study area before final use. Data collectors were given a one-day training by the researchers. The content of training included; purpose of the study, content of the questionnaire in detail, standard interpretation of the questionnaire into the local dialect (Talen), as well as the rights of study participants. Data were checked for completeness and consistency after each day of data collection by holding a meeting with the data collectors. The data were picked from the data collectors at close of every working day.

**Data processing and analysis:** each questionnaire was cross checked again for completeness and consistency before entry. The raw data were entered into Excel and imported into STATA version 13.1 for analysis. Simple proportions and means were used to describe categorical and continuous data respectively. Bivariate analysis was done using Pearson´s Chi-Square and Fisher´s exact test to determine the associations between the independent and dependent variables. The results of the study were presented in the form of tables.

**Measurement:** the dependent variable was menstrual hygiene; measured on a scale of 8 points using the menstrual hygiene management index adopted from Selvi and Ramachandran [19]. A good practice attracted 1 point while poor practice attracted 0 point. The maximum attainable points were 8 and the minimum attainable point was 0. A mean score of 4.95 was then estimated from the total scores of the respondents. A score below 4.95 points was classified as poor menstrual hygiene management while a score of 4.95 points or higher was classified as good menstrual hygiene management.

**Ethical clearance:** the Kwame Nkrumah University of Science and Technology/Komfo Anokye Teaching Hospital Committee on Human Research, Publications and Ethics provided approval for the study. An approval also was sought from the Ghana Education Service. A written informed consent was sought from all the respondents. For all those who agreed to participate in the study, they either signed or thumb printed on the consent form before they were recruited into the study. A witness counter signed or thumb printed. For all those below 18 years, consent was sought from their parents while the students assented to participate in the study. The consenting process included: an explanation of the purpose of the study, confidentiality procedures, benefits, and the freedom to opt out at any time of the study. Respondents were only identified with study codes so as to guarantee confidentiality of the information. Data were kept under lock and key in the office of the researcher while soft copies were password-protected on a computer of the researcher to ensure confidentiality of the data.

## Results

**Menstrual hygiene management index:** a total of 705 girls agreed to participate in the study giving a response rate of 97%. The highest score recorded for menstrual hygiene management (MHM) was 8 points while the least was 2 points with a mean of 4.95±1.92. Almost two-fifths (38.6%) of the total respondents fell below the average score (4.95) and were therefore classified under poor menstrual hygiene management.

**Demographic characteristics of study sample:** menstrual hygiene management in the age categories were similar in terms of those with good and poor menstrual hygiene. A little above half (53.8%) of those who had good menstrual hygiene were in the age category of 16-17 years and a similar proportion (52.6%) of those who managed their menstruation poorly also came from the same age category. Religion was also similar as Christianity dominated with 92.8% and 93% in both good and poor menstrual hygiene groups respectively. Among the good MHM group, 70% of their mothers were non-literate while 82.4% of mothers from the other group were non-literate. When the two groups were compared socio-demographically, (good MHM with poor MHM) they differed significantly in terms of mothers' education level (p=0.0013) and parent socio-economic status (p<0.0001) (Table 1).

**Table 1:** demographic characteristics of participants

Variable	Good MHM (%)	Poor MHM (%)	P-value
**Age group (years)**			**0.9212**
12-15	158 (36.5)	100 (36.7)	
16-17	233 (53.8)	143 (52.6)	
18-19	42 (9.7)	29 (10.7)	
Total	433 (100)	272 (100)	
**Religion**			**0.1116**
Christianity	402 (92.8)	253 (93)	
Islamic	25 (5.8)	10 (3.7)	
Traditional	6 (1.4)	9 (3.3)	
Total	433 (100)	272 (100)	
**Mothers’ education level**			0.0013*
Non-literate	303 (70)	224 (82.4)	
Basic education	109 (25.2)	45 (16.5)	
Secondary education	17 (3.9)	3 (1.1)	
Tertiary education	4 (0.9)	0 (0)	
Total	433 (100)	272 (100)	
**Parent economic status**			**<0.0001**
Low	292 (67.4)	226 (83.1)	
Medium	113 (26.1)	40 (14.7)	
High	28 (6.5)	6 (2.2)	
Total	433 (100)	272 (100)	

N=705. Age group of the participant as well as their religion were fairly distributed among the good and poor MHM but none of those variables was statistically significant. Mother’s education and parent economic status were both statistically significant with MHM at bivariate level.

*Fisher’s exact test

**Self-care practices:** frequent changing of sanitary material was more common among the good MHM group (59.8%) compared to the poor MHM group (16.9%). More than three-fourths (79.8%) of the good MHM group washed their hands with water and soap after changing their sanitary materials whereas only 23.6% of those in the poor MHM group washed their hands with water and soap. Changing of panties once or not at all in a day was very high (91.2%) among the poor MHM group compared with 42% in the good MHM group. All the respondents bathed at least once a day during their menstrual period. The usage of reusable material (sanitary material that are washed, dried and used again) was common (64.7%) among the poor MHM compared with 31.2% in the good MHM. Except “place of drying washable material” that was marginally significant (p-value=0.0521), the two groups differed significantly in all other self-care variables (Table 2).

**Table 2:** self-care practices that participants employ during their menstrual periods

Variable	Good MHM (%)	Poor MHM (%)	P-value
**Sanitary material changes per day**			**<0.0001**
Less than three times	174 (40.2)	226 (83.1)	
Three or more times	259 (59.8)	46 (16.9)	
Total	433 (100)	272 (100)	
**Cleaning of external genitalia**			**<0.0001**
Yes	416 (96.1)	240 (88.2)	
No	17 (3.9)	32 (11.8)	
Total	433 (100)	272 (100)	
**Hand washing after changing sanitary material**			**<0.0001**
Yes	426 (98.4)	225 (82.7)	
No	7 (1.6)	47 (17.3)	
Total	433 (100)	272 (100)	
**Hand washing with soap**			**<0.0001**
Only water	86 (20.2)	172 (76.4)	
Water and soap	340 (79.8)	53 (23.6)	
Total	426 (100)	225 (100)	
**Number of panties changed per day**			**<0.0001**
One or none	182 (42)	248 (91.2)	
Two or more	251 (58)	24 (8.8)	
Total	433 (100)	272 (100)	
**Bath during menstruation**			<0.0001*
Yes	433 (100)	272 (100)	
No	0 (0)	0 (0)	
Total	433 (100)	272 (100)	
**Use of reusable materials**			**<0.0001**
Yes	135 (31.2)	176 (64.7)	
No	298 (68.8)	96 (35.3)	
Total	433 (100)	272 (100)	
**Place of drying washed reusable material**			**0.0521**
Room or under shade	78(58.2)	122 (68.9)	
Outside under sunshine	56 (41.8)	55 (31.1)	
Total	134 (100)	177 (100)	

All the self-care practice variables except 'place of drying washed reusable material' were statistically significant with MHM at bivariate level. The Poor MHM group performed poorly in all the self-care practice variables.

*Fisher's exact test

**Sanitary materials and disposal:** out of the seven hundred and five respondents, four hundred and sixty-four (65.8%) of them used sanitary pads during their last menstruation; the least sanitary material used was toilet tissue, 9 (1.3%). The use of sanitary pad was more common (81.8%) in the good MHM group. More than half (58.9%) of the respondents in the good MHM group said sanitary pads are sold near where they live compared with 43.4% among the poor MHM group. The two groups differed significantly in all the sanitary material and disposal variables. Burial of the used sanitary material was the most practiced method (46.4%) among the good MHM group whereas throwing used pads in open spaces was common among the poor MHM group (47%) (Table 3). Average pad use was 65.8% (not shown).

**Table 3:** sanitary materials used by participants and how they dispose them off

Variable	Good MHM (%)	Poor MHM (%)	p-value
**Type of sanitary material used during last menstruation**			<0.0001*
Sanitary pad	354 (81.8)	110(40.4)	
New cloth	35 (8.1)	66 (24.3)	
Old cloth	40 (9.2)	72 (26.5)	
Cotton	4 (0.9)	15 (5.5)	
Toilet tissue	0 (0)	9 (3.3)	
Total	433 (100)	272 (100)	
**Sanitary pads sold near where you live**			**<0.0001**
Yes	255 (58.9)	118 (43.4)	
No	178 (41.1)	154 (56.6)	
Total	433 (100)	272 (100)	
**Bought pad in the last three months**			**<0.0001**
Yes	222 (51.3)	61 (22.4)	
No	211 (48.7)	211 (77.6)	
Total	433 (100)	272 (100)	
**Reason for not buying pad**			**<0.0001**
I still have some pad	106 (50.2)	34 (16.1)	
I don't have money to buy	105 (49.8)	177 (83.9)	
Total	211 (100)	211 (100)	
**Method of disposing used sanitary materials**			**<0.0001**
Dump in latrine	58 (13.4)	23 (8.5)	
Throw in the open	139 (32.1)	128 (47)	
Bury	201 (46.4)	114 (41.9)	
Burn	35 (8.1)	7 (2.6)	
Total	433 (100)	272 (100)	

The poor MHM group used more reusable material and less sanitary pads, they also had difficulty accessing sanitary pads within where they lived and mostly bury or throw their used sanitary material in the open. All the variables on the table were statistically significant with MHM.

*Fisher's exact test

**Access to sanitary facilities at school:** only one out of the fifteen (15) schools did not have a toilet facility. All the toilet facilities were latrines pits. Two (2) schools had their toilet facilities combined for both girls and boys. Only two (2) of the fourteen schools had water supply at the toilet facilities, meanwhile none had soap for cleaning and washing purposes. Three (3) of the schools had dustbins in the toilets for disposal of used sanitary materials (Table 4).

**Table 4:** availability of sanitary facilities at school

Variable	Frequency (%) N = 14
**Type of toilets**	
Latrine pits	14 (100)
Water closet	0 (0)
**Water in the toilets**	
Yes	2 (14.3)
No	12 (85.7)
**Soap in the toilets**	
Yes	0 (0)
No	14 (100)
**Separate toilet for girls**	
Yes	12 (85.7)
No	2 (14.3)
**Doors in place**	
Yes	10 (71.4)
No	4 (28.6)
**All toilets roofed**	
Yes	12 (85.7)
No	2(14.3)
**Dustbin in the toilets**	
Yes	3 (21.4)
No	11 (78.6)

Fourteen out of the fifteen schools had toilet facilities. However, none of toilet facilities met the minimum requirement for standards menstrual hygiene management.

**School absenteeism:** with regards to school absenteeism, it was more frequent among the poor MHM group (37.1%) compared with the good MHM group (21.5%). On the whole, absenteeism of pupils from school during their menstrual period varied from 1-7 days in a month with a mean of 2.76±1.56 days. Absenteeism from school during menstruation was significantly related to menstrual hygiene management (p-value<0.0001) (Table 5).

**Table 5:** school absenteeism by the type of sanitary material used

Variable	School absenteeism		P-value
**Type of sanitary material used**	Yes (%)	No (%)	0.0001*
Sanitary pad	85 (18.3)	379 (81.7)	
New cloth	41 (40.6)	60 (59.4)	
Old cloth	57 (50.9)	55 (49.1)	
Cotton	7 (36.8)	12 (63.2)	
Toilet roll	4 (44.4)	5 (55.6)	

The type of material used by the participants was statistically significant in relation to school attendance.

*Fisher's exact test

## Discussion

The scores for menstrual hygiene management ranged from a minimum of two to a maximum of eight points. The mean score was 4.95±1.32, which formed the cut off point for good menstrual hygiene management. The study found that 61.4% of the girls practiced good menstrual hygiene. Previous finding from northwestern Nigeria reported that 88.7% of girls in basic schools were engaged in good menstrual hygiene practices [20]. On the other hand, similar studies have found proportions of good menstrual hygiene to be lower than 50% as against this current finding [5,7]. However, our finding is corroborated by the one reported in India where prevalence of good MHM was 63.6% [21]. The disparity in these findings may be due to differences in respondents´ knowledge level, economic background, and socio-cultural factors among others. The age at menarche ranged from 10-16 years with majority of participants falling between 12-15 years age category. This finding is similar to a study conducted by Gumanga and Kwame-Aryee in Accra, Ghana [22]. The mean age at menarche in this study was 13.97±1.17 which is higher than the mean age as found by Gumanga and Kwame-Aryee [22] but lesser than the mean age as reported in a study from Ethiopia [23]. However, the mean age at menarche found as found in our study is similar to the one found in India by Ravi [21]. These variations in age at menarche may be due to the girls´ nutritional status, general health and urbanization which differs from one geographical area to the other.

Parent(s) socio-economic status is said to play a great role in comprehensive menstrual hygiene. This argument is based on the fact that education as well as financial status of parent(s) can influence knowledge and monetary support to girls during menstruation [24]. Mothers´ educational level and parents´ socio-economic status were both significantly associated with menstrual hygiene at bivariate level (p-value=0.0013 and p-value<0.0001 respectively). Mothers who are educated are more likely to provide information to their girls on menstrual hygiene management which may enhance their self-care practices. Also, it is assumed that parents who are economically stable may be able to provide sanitary pads to their menstruating girls as well as provide other basic supplies at home such as water, soap, and privacy which are all necessary for menstrual hygiene management. These findings are corroborated by previous studies [25,26]. Hygiene during menstruation is key to the general wellbeing of the girl and so emphasis is usually placed on the kind of self-care practices that they employ to manage their menses hygienically within recommended standards [17]. In this study, 40.2% of the good MHM group and 83.1% of the poor respondents changed their sanitary materials less than three times in a day, which is below UNICEF recommendation of three or more times in a day [27]. This finding aligns with previous finding from northwestern Nigeria where majority of the school girls changed their sanitary materials only once and at night [20]. Hand washing with water and soap after changing menstrual pads is the standard practice in menstrual hygiene as this will ensure that exposer of menstrual products to others is minimized [17]. Our study found that, hand washing after changing of sanitary material was a common practice; 98.4 % and 82.7% for the good and poor MHM groups respectively. However, 79.8% and 23.6% for the good and poor MHM groups respectively did wash their hands with water and soap. The non-availability of water and soap in the sanitation facilities at schools as found in this study may be a contributory factor to non-washing of hands with water and soap. Bathing during menstruation was observed in all the respondents who participated in the study. On the contrary, findings from Iran, Afghanistan and Lebanon revealed that, menstruating girls were prohibited from bathing for up to three days or more, as a precaution against the risk of excessive blood flow [17,28]. Variation in the findings may be due to differences in socio-cultural practices in different countries.

The current study also found that more than half (64.7%) of the poor MHM group used reusable materials at one point in time and out of this number, majority (68.9%) of them washed and dried their materials in the rooms instead of under the sunshine for proper drying. The washed materials are being dried in the room because these girls feel they may be tease at if they dry them in the open for people to see. This practice may promote the growth of harmful organisms in the materials. Similar findings are reported in previous studies [6,25,26]. The use of sanitary pad was common among rural junior high school girls according to this study. More than half (65.8%) of the respondents used sanitary pads during their last menstruation. This finding is higher than other previous studies conducted in Africa and Asia [7,9,11,13]. It also worth noting that not all the respondents who used sanitary pads in their last menstruation get access to sanitary pad regularly; 17% of them used reusable materials when they run out of sanitary pads. This finding is not different from a previous study by Sudeshna and Aparajita [25].

Our study also found indiscriminate disposal of used sanitary products by the girls; superficial burying or throwing them in open spaces. This finding is in line with a systematic review and meta-analysis by Eijk *et al*. [29] in India where the most common method of disposing used sanitary material among rural girls was burying and indiscriminate throwing. This practice is worrying because the same materials find themselves into water bodies that are being consumed by households [17]. Even those who bury the used materials do so superficially just around their homes. The non-biodegradable ones are turned up into the open during farming season and this equally poses environmental challenges to society [30]. With regards to sanitation facilities, most of the schools had toilet facilities in place, however most of these toilet facilities were not in good condition for the purposes of menstrual hygiene management. This finding agrees with those found by AFAWI [9] in Ghana and Tamiru *et al*. [31] in five African countries. This means that virtually nothing has been done to improve sanitation facilities in schools since the 2011 UNICEF report which indicated that, less than 50% basic schools in developing countries having adequate sanitation facilities. Bodat, Ghate and Majumdar [32] reported that some girls in Pune, India pretend not to be well during their menses due to non-availability of sanitation facilities at school. Sanitation facilities, especially gender specific toilets, are an important component in the management of menstruation among school-going adolescent girls. However, our study found that some of the toilet facilities were combined both girls and boys. School absenteeism was more common among the poor MHM group compared with the good MHM group. On a whole, menstrual related school absenteeism was a little above one-fourth of the respondents. This finding is in line with Sudeshna and Aparajita [25] in India but contrasts those found in northern Ethiopia where school absenteeism was prevalent in more than half (54.5%) of the girls [33]. This discrepancy may be due to variation in available sanitation facilities at school, support by school teachers and availability of sanitary materials among others. The main reasons cited for school absenteeism during menstruation in our study were; menstrual pain, lack of sanitary pads, and fear of staining clothing. Other studies share similar findings [25,33].

**Strength and weakness of the study:** this is the first of its kind to be conducted among basic schools in rural parts of northern Ghana. However, this study lacks qualitative data and was conducted in an area where there was an ongoing free supply of sanitary pads to junior high school girls though the supply was not regular. These findings may not apply to settings where there is no supply of pads.

## Conclusion

This study reveals an average level of menstrual hygiene and self-care practices among school-going adolescent girls in rural settings in northern Ghana. The use of sanitary pads among the respondents was more common than all other sanitary materials and its use was significantly associated with school attendance. Although most of the schools had toilet facilities, they lacked basic requirements such as: water, soap, privacy, and dustbins, which are necessary for menstrual hygiene management. Methods of disposing used sanitary materials were highly unsatisfactory. Mothers' educational level and parents' socio-economic status played a key role in good menstrual hygiene. We recommend government to provide sanitary pads to under-privileged school-going adolescent girls who have attained menarche while advancing steps for local production of standard reusable sanitary materials as a long-term measure. The district assemblies and NGOs should invest more into sanitation facilities at the various schools to reduce school absenteeism due to menstruation.

### What is known about this topic

- Menstruation is associated with school absenteeism and dropout in Ghana;
- The inability of school girls to access sanitary pads is a major challenge to menstrual hygiene management.

### What this study adds

- This study has produced the prevalence of good menstrual hygiene among adolescent girls in rural schools in Ghana [61.4%];
- This study has also demonstrated that, schools in rural northern Ghana lack Water Hygiene and Sanitary (WASH) facilities and this has adversely affected school attendance of girls during their menstrual period;
- Furthermore, the study found that used sanitary materials are improperly disposed in rural parts of northern Ghana.
